# The purpose of peer review in the case of an open-access publication

**DOI:** 10.1186/1750-0680-1-10

**Published:** 2006-09-15

**Authors:** Georgii A Alexandrov

**Affiliations:** 1 Institute of Atmospheric Physics, Russian Academy of Sciences, Pyzhevsky 3, Moscow, Russia

## Abstract

First scientific journals were simply a way of informing colleagues about new research findings. In due course, they started filtering out unreasonable claims, and introduced a peer-review system.

The purpose of peer reviewing changed with time. Since the middle of the past century, commercial publishers have owned a large number of scientific journals and as a result, the marketable value of a submitted manuscript has become an increasingly important factor in publishing decisions.

Recently some publishers have developed business schemes which may stop this tendency. In the case of an open-access publication, the marketable value of a manuscript is not the primary consideration, since access to the research is not being sold. This innovation challenges scientists to re-consider the purpose of peer review.

This editorial indicates some of the commonly used criteria for publication that consequently should receive less or little emphasis under the open-access model.

## Introduction

Many researchers still consider publication in a research journal to be a way of laying claim to a new research result. Many years ago, researchers were doing this by sending mails directly to their colleagues. Then scientific journals appeared. First scientific journals were simply a more efficient way for extensive mailing [1]. In due course they started filtering out unreasonable claims, and introduced the peer-review system.

The purpose of peer reviewing changed with time. Since the middle of the past century, commercial publishers owned a large number of scientific journals and introduced a business scheme that was distinct in nature from the original scheme invented by Henry Oldenburg (see Appendix). In contrast to widespread belief, most commercial scientific publishers did not start out as publishers of scientific journals. They were used to publishing textbooks, not scientific journals, and they started publishing scientific journals as if they were textbooks. This business scheme suggests making a profit from selling research articles to readers – and so the publisher needs to evaluate how many readers will buy an article submitted for publication [2]. Eventually the marketable value of a submitted manuscript became an important factor in publishing decisions.

With the passage of time, the bias has come to be remarkably consistent, and many scientists have forgotten the primary purpose of a scientific publication – to transmit a claim for a new research result to a relevant scientific community. If the claim is reasonable it does not matter whether the readership is large or small [3]. In some sense the purpose of a scientific journal is similar to the purpose of a ship's journal. Keeping an accurate record of scientific achievements is essential in and of itself.

One may hardly expect that a reader-oriented publisher would be willing to pay the cost associated with pursuing such a goal. To be profitable a scientific journal must reject manuscripts that are not found to be sufficiently interesting to a broad readership. Hence, the current business scheme eventually transforms the process of publishing a research result in an unexpected way [4].

Only recently have BioMed Central and some other publishers suggested a business model which may address this problem. In the case of an open-access publication, reviewers need not evaluate the marketable value of a manuscript, since the cost of publication is paid directly (by the author's funder or institution, or by a third party), and does not need to be recouped from readers via subscriptions. This allows publishers to concentrate on the validity of the claim for a new research result – that is, check whether the manuscript is free from obvious errors, or unjustified conclusions.

Under this new model of publishing, scientists can refine the culture of peer-review.

## Discussion

What may be a reason for rejecting an article if the cost of publication does not need to be recouped through subscriptions? In fact, there are many reasons.

First, the article must fall within the general scope of the journal. Second, it must be an original work, not a copy or adaptation of someone else's work. Third, it must show a departure from previous works. Fourth, conclusions must be justified. Moreover, the article must be written in understandable language, and it must contain no obvious errors.

All these requirements are essential for filtering out an unreasonable claim for a new research result and cannot be ignored.

Then, what should not be a reason for rejecting an article, under an open access business model?

Obviously, the marketable value of the article should not be a reason for rejection, and all such evaluations must be ignored.

For example, more than often, peer-review turns into voting. Reviewers simply express their emotions: "interesting"," boring", "good paper", "not impressive", and so on. Such evaluations are essential to predict reader's willingness-to-buy but they are not a primary consideration when choosing what to publish under an open access model.

Some evaluations, however, are not easy to categorize. For instance, reviewers often require revisions to improve manuscript quality. They may ask the author to add more references, or more illustrations, or even to do further research. Most of these recommendations could be really useful to the author as long as they remain discretionary. Nevertheless, if compulsory revisions are required to entertain the reader or to form the impression that the reported results form an entirely solid piece of knowledge, they serve the commercial interests of the publisher, and not that of the research community.

It is worth mentioning in this connection that a journal's reputation is an essential factor for selling it to readers, and therefore publishers commonly confuse textbook science and frontier science. Solid scientific knowledge is the knowledge that has stood the test of time and is well confirmed by a number of independent research studies. Frontier research is that which produces something really new, and something really new cannot be turned into solid knowledge immediately. The vain attempts to transform research journals into instant textbooks may only retard the pace of scientific advances [5].

Evaluations of scientific novelty are also difficult to categorize. There is a tendency to evaluate the "degree of novelty", which is not a well-defined concept. If a manuscript reports something that was not already known, its scientific novelty is obvious. However, the degree of novelty cannot be estimated in an objective manner. (This is again a subjective evaluation, the hidden purpose of which is to evaluate the marketable value of the manuscript.) The tendency to publish only the articles demonstrating a high degree of novelty poses a serious problem. In order to fit such a vague requirement, authors have to exaggerate the significance of their works, which leads, sometimes, to fraud. Since peer review cannot be expected to detect fraud, it would be only prudent to ignore the "degree of novelty" or the "level of interest" [6].

## Conclusion

The obvious advantage of the open-access scheme is that it relieves authors of having to impress readers. It is not clear, however, whether the research community is ready to take advantage of the scheme and re-consider the purpose of peer review, but it is clear that it opens new perspectives, which must be explored.

What makes an open-access journal play a vital role in the research community? Opinions may differ. Some researchers may consider journal's selectivity as a hugely important factor. Therefore, it would be only prudent to address this question to recent and prospective authors of *Carbon Balance and Management*.

Warning: This article does not reflect either the current policy of CBM journal or that of BioMed Central. Its major purpose is to encourage recent and prospective authors to make use of 'Post a comment' tool (see the link on this web page) for exposing their expectations and needs to the members of editorial board, and *vice versa*.

## Appendix. Oldenburg's model of scientific correspondence

Henry Oldenburg is known for creating the first scientific journal – *Philosophical Transactions of the Royal Society of London*. The journal aimed to create a public record of original contributions to knowledge, and set up a new model of scientific correspondence. Let us illustrate the nature of this model by using a case study of a hypothetical research network.

Consider a network of *n *correspondents. Every year each of them sends (*n*-1) copies of a letter informing other network members about obtained research results. The cost of correspondence is, hence, equal to the cost of writing and mailing one copy multiplied by (*n*-1); it grows linearly with the size of network (Figure), and therefore the size of the network is limited by the ability of an average network member to pay this cost.

Oldenburg's model suggests that each correspondent pay the cost of mailing one copy of such a letter and membership fee covering the cost of printing *n *copies of the letter and the cost of delivering *n *copies of it (bound up with other letters) to all network members. The cost of correspondence in this case is not a linear function of network size (Figure), "allowing scientists to reach a wider audience than they would by exchanging private letters" [1].

Nevertheless, the size of the network remains limited by the ability of an average member to pay the membership fee. This may explain why most scientific societies failed to recover publication costs from members' dues since the middle of the past century: the typical size of a scientific society seemingly exceeded a certain threshold. ("The number of U.S. science and engineering Ph.D.'s awarded each year tripled between 1958 and 1968 and continued to increase until the early '70s" [1])

## Competing interests

I am a co-editor of an open-access online journal as well as an author (and co-author) of articles published in traditional journals.

**Figure 1:**
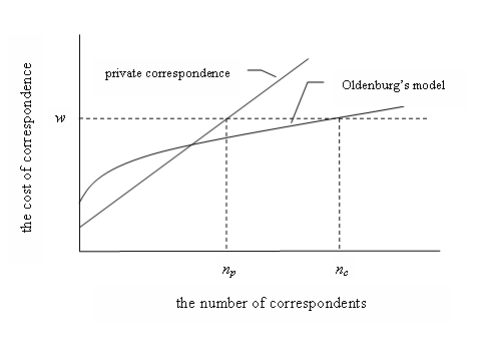
The Oldenburg's model of scientific correspondence. Notation: *w *is paying capacity of an average corresponding member of a research network; *n*_*p *_is maximum size of network based on exchanging private letters; *n*_*c *_is maximum size of network based on Oldenburg's model. .

## References

[B1] Walker TJ (1998). Free Internet Access to Traditional Journals. American Scientist.

[B2] Beschler EF (1998). Pricing of Scientific Publications: A Commercial Publisher's Point of View. Notices Amer Math Soc.

[B3] Wager E (2006). Ethics: What is it for?. Nature: Web Debate – Peer-review.

[B4] Guedon J-C (2001). In Oldenburg's Long Shadow: Librarians, Research Scientists, Publishers, and the Control of Scientific Publishing.

[B5] Wade N Lowering Expectations at Science's Frontier. New York Times.

[B6] Bloom T (2006). Systems: Online frontiers of the peer-reviewed literature. Nature: Web Debate – Peer-review.

